# Characterization and complete genome of the virulent *Myoviridae* phage JD007 active against a variety of *Staphylococcus aureus* isolates from different hospitals in Shanghai, China

**DOI:** 10.1186/s12985-017-0701-0

**Published:** 2017-02-08

**Authors:** Zelin Cui, Tingting Feng, Feifei Gu, Qingtian Li, Ke Dong, Yan Zhang, Yongzhang Zhu, Lizhong Han, Jinhong Qin, Xiaokui Guo

**Affiliations:** 1 Department of Immunology and Microbiology, School of Medicine, Shanghai Jiao Tong University, Shanghai, 200025 China; 2 Department of Laboratory Medicine, Shanghai General Hospital, Shanghai Jiao Tong University School of Medicine, Shanghai, 200080 China; 3 Department of Clinical Pharmacy, Shanghai General Hospital, Shanghai Jiao Tong University School of Medicine, Shanghai, 200080 China; 4 Department of Clinical Microbiology, Shanghai Ruijin hospital, Shanghai, 200025 China; 5 Department of Laboratory Medicine, Ruijin Hospital, Shanghai Jiao Tong University School of Medicine, Shanghai, 200025 China

**Keywords:** *Staphylococcus* phage JD007, MRSA, Host-range

## Abstract

**Background:**

The implementation of phage therapy is re-emerging with the increase in widespread antibiotic-resistant bacteria.

**Methods:**

*Staphylococcus* phage JD007 was characterized and its complete genome sequence analysed.

**Results:**

*Staphylococcus* phage JD007 was classified as belonging to the *Myoviridae* family based on its morphology, as observed by transmission electron microscopy. Its lytic activity was stable between pH 5–11 and below 42 °C; moreover, an absorbance curve showed that nearly 90% of the viral particles had adsorbed to its host after a 20 min co-incubation. The complete genome size is 141,836 bp, making JD007 one of the largest *Staphylococcus* phages of *Myoviridae*. No identifiable resistance or virulence genes were found in the JD007 genome. JD007 was able to lyse 95% of *S. aureus* isolates, including the prevalent ST239-MRSA and ST59-MRSA strains isolated from different hospitals in Shanghai, China, and inhibition assays showed that JD007 could inhibit *S. aureus* growth at a multiplicity of infection of 0.1.

**Conclusions:**

The results suggested that *Staphylococcus* phage JD007 can potentially be used in phage therapy or for the detection of *S. aureus*.

**Electronic supplementary material:**

The online version of this article (doi:10.1186/s12985-017-0701-0) contains supplementary material, which is available to authorized users.

## Background

*Staphylococcus aureus* is considered a commensal and major human pathogen responsible for a variety of acute and chronic diseases [1]. Due to the broad range of methicillin-resistant *S. aureus* (MRSA) strains and the emergence of vancomycin- resistant *S. aureus* (VRSA) strains [2], it is feasible that in the future, there will be a lack of antibiotics available to treat antibiotic-resistant infectious diseases. Accordingly, there is recent renewed interest in phage therapy. Indeed, phages that infect *S. aureus* have been used in clinical trials to treat chronic venous leg ulcers, and the results show no safety concerns with regard to the use of bacteriophage treatment [3]. Therefore, *Staphylococcus* phages can potentially be used for phage therapy.

*Staphylococcus* phages belonging to the *Myoviridae* family have been isolated worldwide [4–8]. The phages Romulus and Remus are able to infect approximately 70% of the tested *S. aureus* isolates and display lytic activity inside these hosts. Furthermore, both phages exhibited rapid initial adsorption and biofilm-degrading capacity [9]. In one study, phage ΦSA039 produced clear plaques on 13 of 15 *Staphylococcus* isolates (87%) [10], and phage Stau2 lysed 80% of the *S. aureus* isolates (164/205) obtained from hospitals in Taiwan, China [11]. Furthermore, phage ISP successfully infected 86% (31/34) of *Staphylococcus* isolates in another study, including relevant MRSA strains [12], and phage MSA6 infects a wide spectrum of staphylococcal strains originating from both humans and bovines [13]. In summary, these studies suggest that phages belonging to *Myoviridae* can kill a broad range of *S. aureus*.

In this paper, a *Staphylococcus* phage named JD007 was isolated from chicken faeces in Shanghai, China. Its morphology was assessed using transmission electron microscopy, its thermal and pH stability were evaluated, the inhibition assays and host adsorption rate were characterized, and the complete genome was sequenced and analysed. Furthermore, the host range of JD007 was characterized using prevalent strains of *S. aureus* isolated from different hospitals in Shanghai, China, to characterize JD007 *in vitro* bactericidal activity.

## Results and Discussion

### Antimicrobial susceptibility testing, MLST sequence types (STs), and spa typing of *S. aureus*

A total of 175 strains of *S. aureus* were obtained from different hospitals in Shanghai, China. As shown in Additional file 1, all 175 isolates were susceptible to vancomycin, teicoplanin, and linezolid. Regarding other antibiotics, the following results were observed: 8/175 were susceptible to penicillin; 54/175 were susceptible to gentamycin; 1/175 were intermediate susceptible to gentamycin; 35/175 were susceptible to oxacillin; 38/175 were susceptible to erythromycin; 6/175 were intermediate susceptible to erythromycin; 60/175 were susceptible to clindamycin; and 103/175 were susceptible to rifampicin. The strains can be divided into several different multilocus sequence (MLS) and *Staphylococcus* protein A gene (Spa) types, including 55/175 belonging to the ST239-t030 type, 28/175 belonging to the ST239-t037 type, 18/175 belonging to the ST764-t002 type, and 13/175 belonging to the ST641-t067 type. ST1-t127, ST217-t309, ST398-t034, ST5-t002, ST59-t441, and ST88-t2310 types were also present.

### *Staphylococcus* phage JD007 morphology

**Fig. 1:**
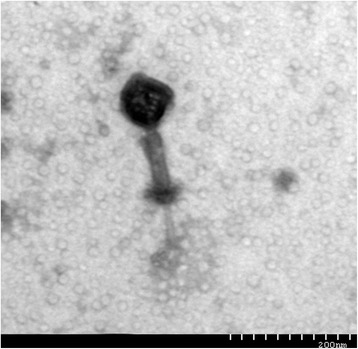
The morphology of *Staphylococcus* phage JD007. This photo was obtained by transmission electron microscopy. As shown, the head diameter of *Staphylococcus* phage JD007 is approximately 60 nm; the length of its tail is approximately 100 nm, and a contractile sheath between the head and tail is present. Scale bar, 200 nm

A photograph of the bacteriophage JD007 was obtained by transmission electron microscopy, as shown in Fig. 1. Its head is approximately 60 nm in diameter, the length of its tail is approximately 110 nm, and the contractile sheath can be observed between the head and tail. Based on these morphological features, the phage belongs to *Myoviridae. S. aureus* phages belonging to *Myoviridae* have been isolated worldwide, including phages K, SA5, A5W, Sb-1, ISP, G1, GH15, SA11, Staphy1N, MSA6, 676Z, P4W, Fi200w, vB_SauM_Remus, vB_SauM_Romulus, Twort, phiIPLA-RODI, and phiIPLA-C1C [4–8]. Several studies have shown that these phages can prevent infections in animal models. JD007 was isolated from chicken faeces in Shanghai, China. These *Myoviridae* phages have genome sizes between 120 and 140 kb, exhibit a G + C content of 27.98 to 30.60%, and encode 183 to 217 open reading frames (ORFs).

### Biophysical stability infection parameters

**Fig. 2:**
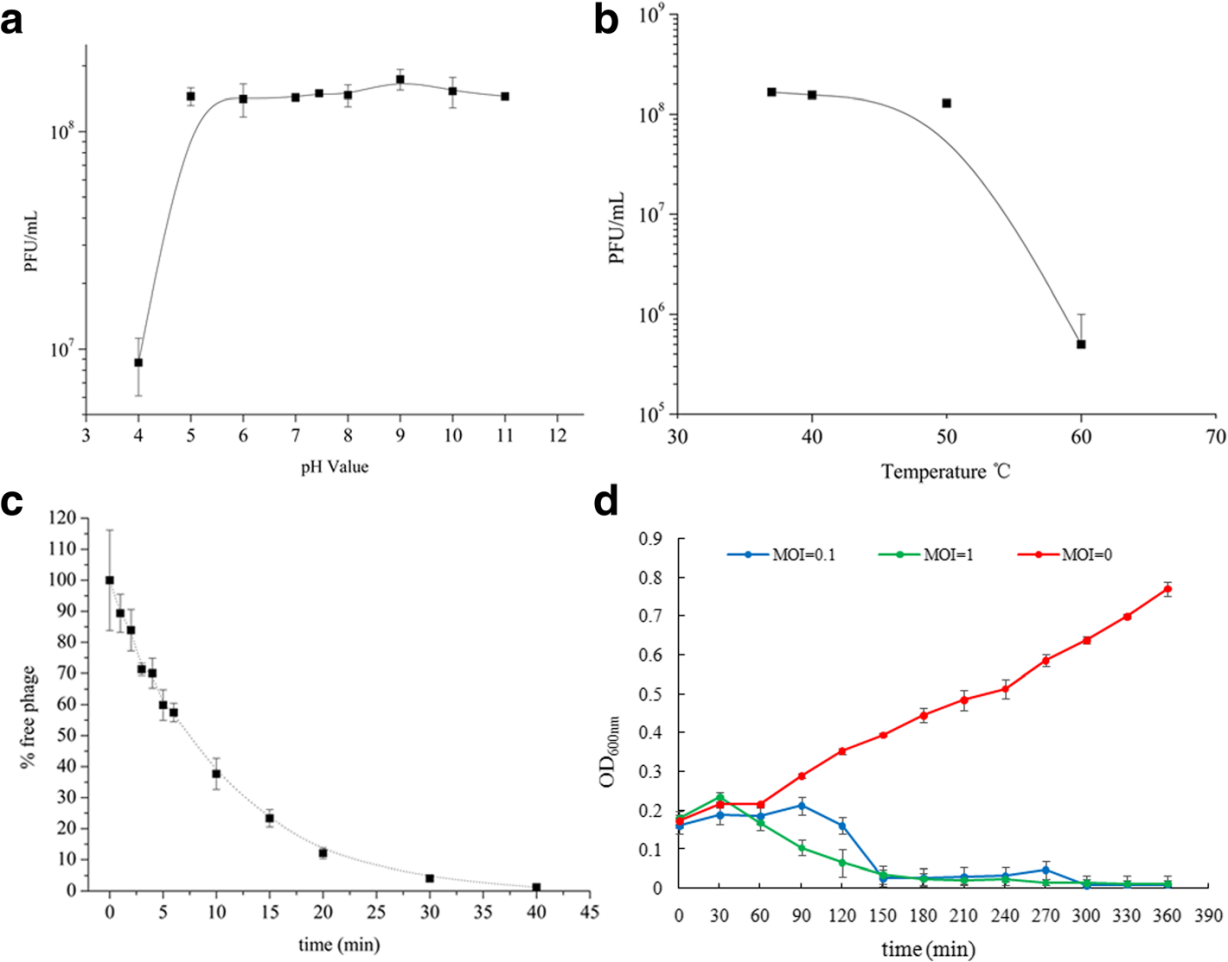
Characterization of *Staphylococcus* phage JD007. **a** The pH stability of *Staphylococcus* phage JD007, with the x-axis representing different pH values, % stability with reference pfu/mL at pH 7.45. **b** The x-axis represents different temperatures, and the y-axis represents the relative titres of *Staphylococcus* phage JD007 compared to that at 37 °C. % stability = (N/N_0_) × 100, where N is the number of viable phages after 1 h of incubation and N_0_ is the initial number of phages. **c** Inhibition assays of *Staphylococcus* phage JD007. The x-axis represents the co-culture time of *Staphylococcus* phage JD007 and Sa60, and the y-axis represents the change in OD_600_ in the mixture of *Staphylococcus* phage JD007 infecting its host at different MOIs. The red, blue and green circles represent MOI = 0, 0.1 and 1, respectively. **d** The adsorption rate of *Staphylococcus* phage JD007 to its host *S. aureus* Sa60, with the x-axis representing the incubation time of *Staphylococcus* phage JD007 and its host, and the y-axis represents the percent of titres of phage that had not been absorbed to the host. The error bars represent the s.d

The stability of phage JD007 was also evaluated. Lytic activity was stable between pH5 and 11 (Fig. 2a), whereas activity was completely lost when kept below pH 4 for 2 h at room temperature. As shown in Fig. 2b, the activity of phage JD007 was stable between 37 and 42 °C, however, when kept at 50 °C for 1 h, activity decreased by 25% compared to 37 °C. Furthermore, JD007 lost nearly all activity at a temperature above 60 °C. *Sangeeta et al.* also have reported a lytic phage of *Salmonella enterica* serovar Paratyphi B that could keep viral activity stable between pH 4 and 9 or between 4 and 40 °C [14]. Based on thermal and acid stability tests, phage JD007 remained stable at room temperature and between pH 5 and 11.

### The adsorption rate of *Staphylococcus* phage JD007

We also sought to characterize the adsorption rate of phage JD007 to its host cell, as shown in Fig. 2d. Phage JD007 adsorbed to its host cell *S. aureus* Sa60 80, 90, and 97% of the time when incubated at 37 °C for 15, 20, and 30 min, respectively.

### Inhibition assays of *Staphylococcus* phage JD007

To identify the *in vitro* bactericidal activity of phage JD007, *S. aureus* Sa60 was cultured at an OD_600_ of 0.2 and then infected with phage JD007 at a multiplicity of infection (MOI) of 0, 0.1 and 1 at 37 °C with shaking at 100 rpm. As shown in Fig. 2c, phage JD007 inhibited the growth of *S. aureus* after co-culture for 150 min, whereas *S. aureus* Sa60 was able to grow normally without phage JD007. Our results suggest that *Staphylococcus* phage JD007 can be used for inhibition the growth of *S. aureus*. Gutierrez reported that *Staphylococcus* phages phiIPLA-RODI and phiIPLA-C1C had the same lytic activity on *S. aureus* IPLA16, since no viable bacteria were detected after 8 h of incubation and a considerable decrease of the bacterial population was already achieved after 6 h of treatment [15].

### The host range of *Staphylococcus* phage JD007

A total of 175 *S. aureus* isolates from different hospitals in Shanghai, China, were chosen to identify the host range of *Staphylococcus* phage JD007. Our results showed that phage JD007 could kill 95% (166/175) of *S. aureus* isolates of different types. As shown in Table 1, 41 of the 175 isolates representing different types were identified and characterized through MLS and Spa typing. *Staphylococcus* phage JD007 formed an inhibition zone in a double-layer plate, some of which were clear and some faint. *Staphylococcus* phage JD007 formed clear inhibition zones using ST239-t030 that were isolated from different hospitals in Shanghai, China. Importantly, phage JD007 killed the prevalent strains of ST239-t030 that account for an increase in the incidence of infectious diseases [16]. Phage JD007 also killed other types of isolates, including ST1-t127, ST1128-t164, ST188-t189, and ST239-t459, with high efficiency. Furthermore, phage JD007 killed the majority of ST239-t030 and ST59-MRSA strains, with different efficiencies, though several strains of ST239-t030 were absolutely not susceptible to phage JD007. We suggest that JD007 has a wide host range with different lytic efficiencies. Furthermore, no evidence for a significant correlation between phage activity and antimicrobial susceptibility, ST, or Spa type was found because phage JD007 killed different strain types with different antimicrobial susceptibilities at variable efficiencies.

**Table 1:** The host range of *Staphylococcus* phage JD007

Sample No.	ST	SCCmec	spa	Phage JD007
S130069	ST1		t127	++++
S130089	ST1281		t164	++++
S130192	ST1301		t12145	++++
S130099	ST15		t084	+++
S130102	ST1281		t377	+
S130066	ST188		t189	++++
S130188	ST20		t164	++++
S130077	ST217		t309	++++
S130174	ST2315		t11687	++++
S130060	ST239	III	t030	++++
S130078	ST239	III	t037	+++
S130164	ST239	III	t298	++
S130103	ST239	III	t459	++++
S130091	ST239		t030	++
S130084	ST25		t078	++++
S130072	ST398		t034	++++
S130061	ST398		t1255	++++
S130098	ST398		t571	++++
S130086	ST5	II	t002	++++
S130095	ST5	II	t010	+++
S130076	ST5		t002	++++
S130173	ST5		t570	++++
S130065	ST5		t954	++++
S130193	ST59	IV	t163	+++
S130096	ST59	IV	t172	++++
S130158	ST59	IV	t437	+++
S130064	ST59	IV	t441	++++
S130177	ST59	IV	t163	+
S130166	ST59		t437	++++
S130100	ST6		t701	++++
S130183	ST630	V	t5554	+++
S130178	ST630		t12148	++
S130162	ST630		t377	+++
S130087	ST7	NT	t12146	++++
S130059	ST7		t091	++++
S130105	ST7		t6248	++++
S130063	ST7		t796	++++
S130088	ST88	IV	t2310	++++
S130070	ST88	NT	t12147	+++
S130071	ST88		t2310	+++
S130181	ST965		t062	+++

(The “+” represent the activity of phage JD007 to its correspondence hosts, the more “+” the more clear inhibition zones were formed by the phage JD007)

### Safety assessment of *Staphylococcus* phage JD007 based on the genome sequence

**Fig. 3:**
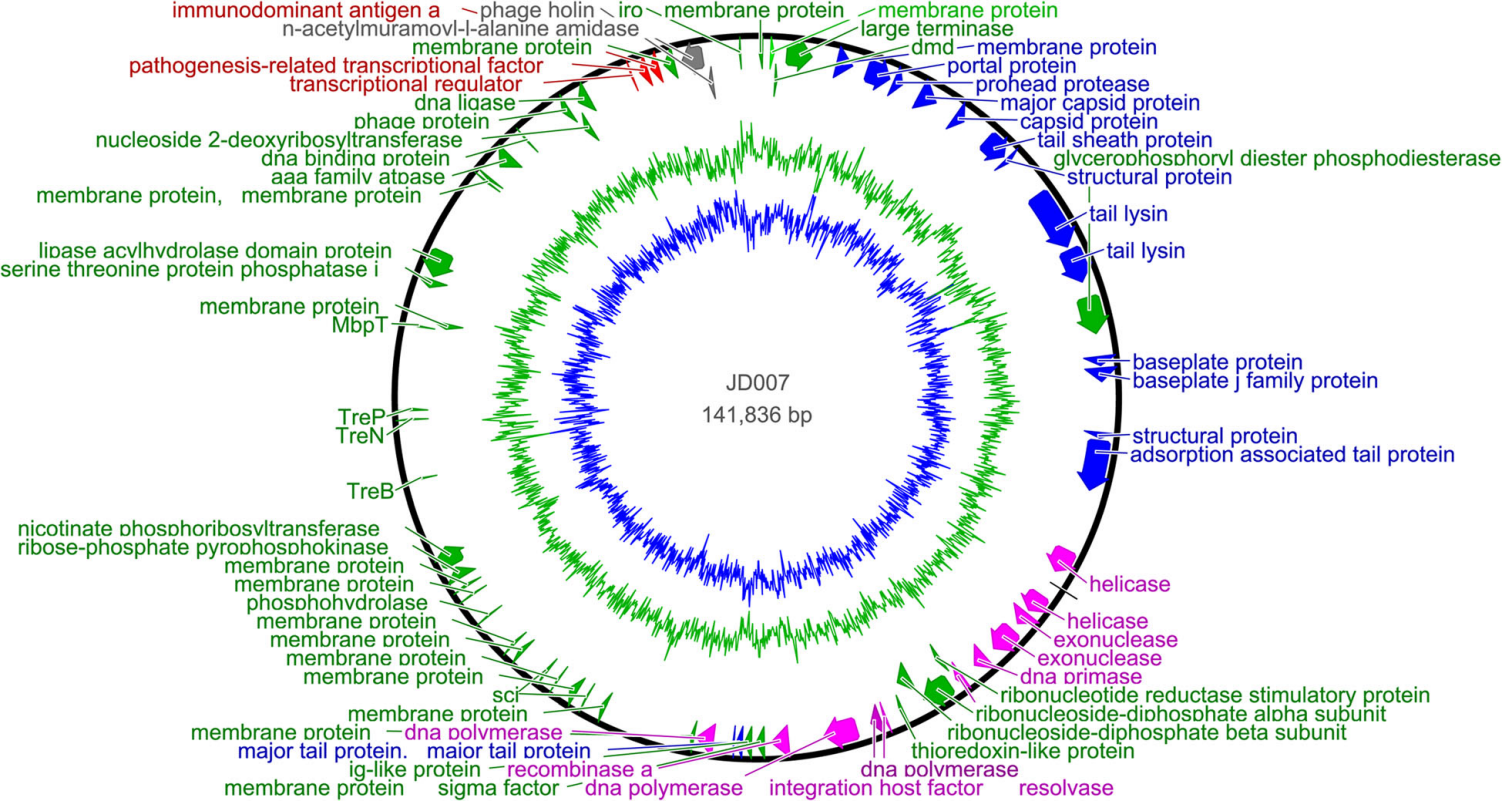
The genome structure of *Staphylococcus* phage JD007. The complete genome of *Staphylococcus* phage JD007 is 141,836 bp and contains 217 ORFs. The arrows represent predicted ORFs

**Fig. 4:**
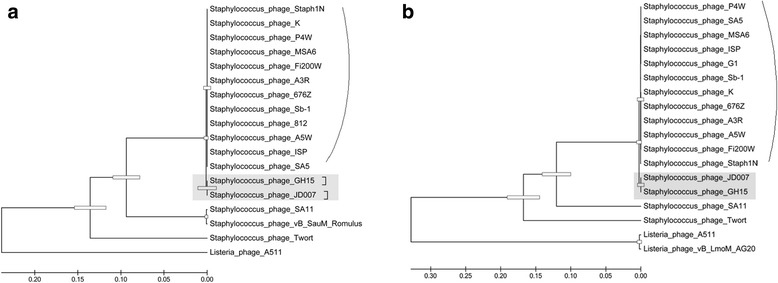
The phylogenetic tree of bacteriophages of *Myoviridae*. **a** and **b** separately represented the maximum likelihood tree constructed using the proteins of major capsid protein and helicase encoded encoded in their genome

**Fig. 5:**
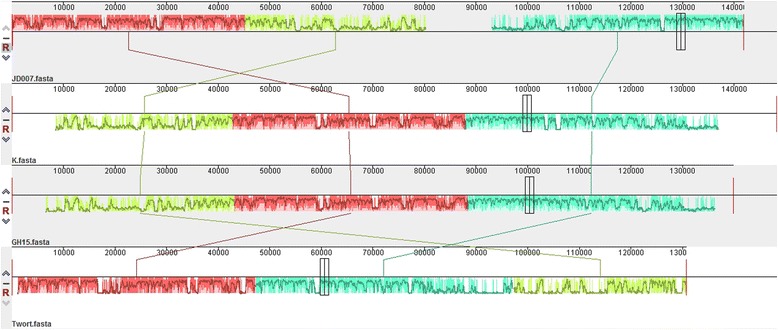
Comparative genomic analysis of *Staphylococcus* phage JD007 with phages K, Twort and GH15 that belong to *Myoviridae*

The complete genome of *Staphylococcus* phage JD007 is 141,836 bp, and contains 217 ORFs [17]. The entire genome structure of *Staphylococcus* phage JD007 is shown in Fig. 3; the arrows represent the predicted ORFs consisting of genes involved in the bacteriophage structure and in DNA replication as well as other predicted functions. The proteins of helicase and major capsid protein were separately used to draw phylogenetic trees, as showed in Fig. 4a and Fig. 4b; phage JD007 and other *Staphylococcus* phages belong to *Myoviridae* were clustered in the same branch of tree, these results were constant with the results of its morphology. The phages chosen for phylogenic analysis are all belonged to *Myoviridae*, their host are different: phage A511 infects *Listeria monocytogenes* [18], while others infect *S. aureus*. Phage GH15 and JD007 were separately isolated from Jilin province and Shanghai, China, there are more than 1000 km between these two places, these two phages were clustered in the same sub-branch, it meant their closest genetic relationship. All of the ORFs predicted by Glimmer were searched by blast in the database of toxin and antibiotic resistance genes, with no identifiable toxin or antibiotic resistance genes found in the genome until present. While, phage genomes are replete with genes of unknown function and it is possible that the genome of this phage contains toxin or resistance genes, they may be just of types that have not yet been characterized until present, so it is necessary to systematically study these genes’ function for fully understanding safety of the phage for therapy. The complete genomic comparisons of phage JD007 with phage K, Twort and GH15 were showed in Fig. 5, its coverage of GH15 was 93% with identity of 97%, while its coverage of K was 85% with identity of 98%, and its coverage of Twort was 74% with identity of 8%. The results indicated the mosaic genomic structure among these bacterophages.

Bacteriophages can be considered vectors that transmit antibiotic resistance genes or virulence genes amongst bacteria. The prophage may contribute the pathogenic traits of *Enterococcus faecalis* [19]. Antibiotic resistance genes, such as blaTEM, qnrA, and blaCTX-M-1, as well as toxin genes, have been found in the genomes of bacteriophages isolated from human faecal samples [20, 21]. Thus, the predicted ORFs of *Staphylococcus* phage JD007 were searched using blast against the database of virulence factors and antibiotic resistance genes, and no virulence or antibiotic resistance genes were found until present. This indicates that *Staphylococcus* phage JD007 will likely not act as a vector of dissemination of such undesired genes amongst bacteria when used for the prevention or control of *S. aureus*.

## Conclusion

*Staphylococcus* phage JD007 is stable at room temperature and under acidic conditions. It can quickly adsorb to its host cell and propagate in that host cell and has a wide host range. In addition, no antibiotic resistance or toxin genes were found in its genome until present. The phage kills 95% of *S. aureus* strains of different types, and most importantly, it can kill ST239-MRSA III-Spa t030 strains, which are prevalent and accounts for the increase in infectious disease incidence in China. JD007 may be potentially useful for phage therapy or the detection of *S. aureus* clinically.

## Methods

### Bacteria isolates and culture conditions

A total of 175 isolates of *S. aureus* were obtained from Ruijin Hospital, the Sixth People’s Hospital of Shanghai, the Armed Police General Hospital, and the Centre Hospital of Changning District in Shanghai, China. The strains isolated from Ruijin Hospital were kindly provided by Qingtian Li, Those from the Sixth Hospital by FeifeiGu and Lizhong Han, those from the Armed Police General Hospital by Yunheng Zhou, and those from the centre hospital of Changning District in Shanghai, China were by Ren Wang. The use of these isolates in this paper was approved by these individuals, the ownership of the strains individually belonged to them, and we were authorized to use these isolates in this paper. The isolates were grown in liquid LB (Luria-Bertani) medium at 37 °C, on solid LB medium (1.5% agar), or in LB soft agar overlays (0.7% agar). Phage JD007 was isolated from chicken faeces collected from the chicken slaughter facility in the Madang food market located on No.349-2# Madang Road in the Huangpu District of Shanghai, China [17]. *S. aureus* strain Sa60 isolated from Ruijin Hospital was used for phage JD007 amplification and the following experiments. Forty-one strains of the 175 total strains represent different types of *S. aureus* identified and characterized using MLS and Spa typing methods, as described previously [22]. Briefly, the strains were spa-typed via the online database (http://www.spaserver.ridom.de/). The sequence type (ST) was characterized by multi-locus sequence typing (MLST), and the products of seven house-keeping gene fragments were sequenced (Sangon Biotech, Shanghai) and compared to allele profiles from the database of *S. aureus* (http://saureus.mlst.net/). All of the bacteria were identified using the VITEK2 compact system, and antimicrobial susceptibility testing was performed by the disk diffusion method according to Clinical and Laboratory Standards Institute guidelines (M100S, 26^th^edition) for penicillin (P) (10 units), cefoxitin (30 μg), gentamicin (CN) (10 μg), erythromycin (E) (15 μg), teicoplanin (30 μg), clindamycin (CC) (2 μg), rifampicin (5 μg), and linezolid (30 μg). The minimum inhibitory concentration (MIC) of vancomycin was determined using an E-test. *S. aureus* ATCC25923 and ATCC29213 were used as quality controls for the disk diffusion test and MIC detection, respectively.

### Bacteriophage amplification and purification

High-titre phage stocks were obtained through amplification in liquid LB medium containing 10 μM MgCl_2_ and 5 μM CaCl_2_. First, phage JD007 that infected *S. aureus* Sa60 cells at an MOI of 0.1 was incubated at 37 °C overnight. Visible lysis of the liquid culture was obtained, and the lysate was then incubated with chloroform (final concentration of 2%) for 30 min with gentle shaking to kill the remaining bacteria. Bacteria debris was removed by centrifugation at 6,500 rpm (Beckman, JA18.0, USA) for 15 min. The phages in the supernatant were enriched at 4 °C overnight using polyethylene glycol (PEG) 8000 and precipitated (final concentration 10%w/v) at 8,500 rpm for 20 min (Beckman, JA18.0). The pellet was dissolved in TM buffer and vortexed. PEG8000 was removed after adding the same volume of chloroform after vortexing, and the solution was centrifuged at 4,000 × g for 10 min, the supernatant contained a high concentration of phage. CsCl was added at a concentration of 0.5 g per 1 mL, and the phages were purified by discontinuous centrifugation through a CsCl gradient (1.33, 1.45, 1.50, and 1.70 g/cm^3^) in TM buffer in Ultra-Clear tubes (Beckman Coulter, Inc., Fullerton, CA) at 120,000 × g for 4 h. The band of enriched phages was removed using a syringe, and the same was dialyzed against TM buffer and stored at 4 °C.

### Electron microscopic imaging

Using the purified phage obtained above. Then, phage particles were collected by centrifugation at 33,000 × g for 1 h and washed twice in 0.1 × PBS (pH7.4) using a Beckman high-speed centrifuge and a JA-18.1 fixed-angle rotor. Following deposition onto a carbon-coated copper grid and staining with 2% (wt/vol) potassium phosphotungstate (pH 7.0), the grids were observed using a Hitachi H7500 transmission electron microscope (TEM) operating at 80 kV.

### Phage physical characterization

#### The stability of *Staphylococcus* phage JD007

The acid–base stability of phage JD007 was assessed. One hundred-fold dilutions of the initial phage titre (10^8^ pfu/mL) were performed using TM buffer at different pH values ranging from 2 to 11, followed by a 2-h incubation at 37 °C. Several samples were adequately diluted, and 50 μL was collected to assess the phage titre using the double-layer plate method.

For the thermal stability assessment of phage JD007, a titre of 10^8^ pfu/mL was incubated separately at different temperatures (37, 42, 50, 60, 70 and 80 °C) for 1 h, The titre of JD007 at different temperatures was confirmed using the double-layer plate method with *S. aureus* Sa60 cultured to an OD_600nm_ ≈ 0.4.

#### Adsorption rate of *Staphylococcus* phage JD007 to Sa60

Phage JD007 and *S. aureus* Sa60 were incubated at an MOI of 0.01 for 1, 2, 3, 4, 5, 6, 10, 15, 20, 30, or 40 min; the mixtures were centrifuged at 16,000 × g for 30 s. The titre of phage in the supernatant was identified using the double-layer agar plate method. The titre of phages previously mixed with *S. aureus* Sa60 was considered time “0”. The proportion of the amount of non-adsorbed phages to the amount of phages used for infection, based on three independent experiments, is shown, and standard deviations are indicated.

#### The inhibition assays of *Staphylococcus* phage JD007 to *S. aureus* Sa60

*S. aureus* Sa60 was cultured to an OD_600nm_ ≈ 0.2 and then infected by phage JD007 at an MOI of 0, 0.1, or 1. The mixtures were incubated at 37°Cwith shaking at 100 rpm, and the OD_600nm_ value at different time points was measured using a BioPhotometer plus.

### The host range of *Staphylococcus* phage JD007

The host range was analysed by spotting serial dilutions of phage JD007 on a double-layer soft agar lawn of different *S. aureus* isolates obtained from different hospitals. Two microlitres of concentrated phage lysate (≈10^8^ pfu/mL) and serial dilutions were plated onto LB medium plates overlaid with *S. aureu*s (OD_600nm_ ≈ 0.4) mixed with 0.7% top agar (cultured for 30 min before adding), followed by overnight incubation. The strains with the same infectivity as the control strain (*S. aureus* Sa60) have an efficiency of plating of 1 (probably the equivalent of your “++++”). The inhibition zone of the spots formed by spot testing were seen as a common system for assessing the success of infection by the phage: ++++ complete clearing; +++ clearing throughout but with faintly hazy background; ++ substantial turbidity throughout the cleared zone; + a few individual plaques; 0 no clearing, − but you may see a spot where the pipette tip touched the agar [23]. In total, 175 isolates of *S. aureus* were used to identify the host range of phage JD007.

### Safety assessment of *Staphylococcus* phage JD007 based on the genome sequence

The complete genome sequence of JD007 has been reported, GenBank accession number is JX878671 [17]. It was sequenced using Roche 454 Sequencing Technology and assembled using the method of Newbler Metrics Results Software Release v. 2.7 the platform provided. All of the annotated genes were compared to an antibiotic resistance gene database (ARDB, http://ardb.cbcb.umd.edu/) and a virulence factor database (http://www.mgc.ac.cn/VFs/main.htm). Genes with more than 70% coverage and 30% identity were retained. The comparison of complete genome sequences of phage JD007 with phage K [24], Twort [4] and GH15 [25] using Mauve20150226 [26]. The maximum likelihood trees were constructed using MEGA5 [27].

## Additional file

Additional file 1: The details of S.aureus chosen for experiments. (XLSX 32 kb)

## Abbreviations

BLAST: Basic local alignment search tool
ORFs: Open reading frames.
PFU: Plaque forming unit, a measure of the number of viable viral particles

## References

[1] Otto M (2010). Basis of virulence in community-associated methicillin-resistant *staphylococcus aureus*. Annu Rev Microbiol.

[2] Deen J, von Seidlein L, Andersen F, Elle N, White NJ, Lubell Y (2012). Community-acquired bacterial bloodstream infections in developing countries in south and southeast Asia: a systematic review. Lancet Infect Dis.

[3] Rhoads DD, Wolcott RD, Kuskowski MA, Wolcott BM, Ward LS, Sulakvelidze A (2009). Bacteriophage therapy of venous leg ulcers in humans: results of a phase I safety trial. J Wound Care.

[4] Kwan T, Liu J, DuBow M, Gros P, Pelletier J (2005). The complete genomes and proteomes of 27 *staphylococcus aureus* bacteriophages. Proc Natl Acad Sci U S A.

[5] Gu J, Liu X, Yang M, Li Y, Sun C, Lu R, Song J, Zhang Q, Lei L, Feng X (2013). Genomic characterization of lytic *staphylococcus aureus* phage GH15: providing new clues to intron shift in phages. J Gen Virol.

[6] Gutierrez D, Vandenheuvel D, Martinez B, Rodriguez A, Lavigne R, Garcia P (2015). Two phages, phiIPLA-RODI and phiIPLA-C1C, lyse mono- and dual-species staphylococcal biofilms. Appl Environ Microbiol.

[7] Kim MS, Myung H (2012). Complete genome of *staphylococcus aureus* phage SA11. J Virol.

[8] Lobocka M, Hejnowicz MS, Dabrowski K, Gozdek A, Kosakowski J, Witkowska M, Ulatowska MI, Weber-Dabrowska B, Kwiatek M, Parasion S (2012). Genomics of staphylococcal twort-like phages--potential therapeutics of the post-antibiotic era. Adv Virus Res.

[9] Vandersteegen K, Kropinski AM, Nash JH, Noben JP, Hermans K, Lavigne R. Romulus and Remus, two phage isolates representing a distinct clade within the Twortlikevirus genus, display suitable properties for phage therapy applications. J Virol. 2013;87:3237–3247. 10.1128/JVI.02763-12 PMC3592175 23302893

[10] Synnott AJ, Kuang Y, Kurimoto M, Yamamichi K, Iwano H, Tanji Y (2009). Isolation from sewage influent and characterization of novel *staphylococcus aureus* bacteriophages with wide host ranges and potent lytic capabilities. Appl Environ Microbiol.

[11] Hsieh SE, Lo HH, Chen ST, Lee MC, Tseng YH (2011). Wide host range and strong lytic activity of *staphylococcus aureus* lytic phage Stau2. Appl Environ Microbiol.

[12] Vandersteegen K, Mattheus W, Ceyssens PJ, Bilocq F, De Vos D, Pirnay JP, Noben JP, Merabishvili M, Lipinska U, Hermans K, Lavigne R (2011). Microbiological and molecular assessment of bacteriophage ISP for the control of *staphylococcus aureus*. PLoS One.

[13] Kwiatek M, Parasion S, Mizak L, Gryko R, Bartoszcze M, Kocik J (2012). Characterization of a bacteriophage, isolated from a cow with mastitis, that is lytic against *staphylococcus aureus* strains. Arch Virol.

[14] Ahiwale SS, Bankar AV, Tagunde SN, Zinjarde S, Ackermann HW, Kapadnis BP (2013). Isolation and characterization of a rare waterborne lytic phage of *salmonella enterica* serovar paratyphi B. Can J Microbiol.

[15] Gutierrez D, Vandenheuvel D, Martinez B, Rodriguez A, Lavigne R, Garcia P. Two Phages, phiIPLA-RODI and phiIPLA-C1C, Lyse Mono- and Dual-Species Staphylococcal Biofilms. Appl Environ Microbiol. 2015;81:3336–3348. 10.1128/AEM.03560-14 PMC4407228 25746992

[16] Zhou W, Shan W, Ma X, Chang W, Zhou X, Lu H, Dai Y (2012). Molecular characterization of rifampicin-resistant *staphylococcus aureus* isolates in a Chinese teaching hospital from Anhui. China BMC Microbiol.

[17] Cui Z, Song Z, Wang Y, Zeng L, Shen W, Wang Z, Li Q, He P, Qin J, Guo X (2012). Complete genome sequence of wide-host-range *staphylococcus aureus* phage JD007. J Virol.

[18] Loessner MJ, Scherer S (1995). Organization and transcriptional analysis of the *Listeria* phage A511 late gene region comprising the major capsid and tail sheath protein genes cps and tsh. J Bacteriol.

[19] Matos RC, Lapaque N, Rigottier-Gois L, Debarbieux L, Meylheuc T, Gonzalez-Zorn B, Repoila F, Lopes Mde F, Serror P (2013). *Enterococcus faecalis* prophage dynamics and contributions to pathogenic traits. PLoS Genet.

[20] Quiros P, Colomer-Lluch M, Martinez-Castillo A, Miro E, Argente M, Jofre J, Navarro F, Muniesa M (2014). Antibiotic resistance genes in the bacteriophage DNA fraction of human fecal samples. Antimicrob Agents Chemother.

[21] Martinez-Castillo A, Quiros P, Navarro F, Miro E, Muniesa M (2013). Shiga toxin 2-encoding bacteriophages in human fecal samples from healthy individuals. Appl Environ Microbiol.

[22] Gu FF, Han LZ, Chen X, Wang YC, Shen H, Wang JQ, Tang J, Zhang J, Ni YX (2015). Molecular characterization of *staphylococcus aureus* from surgical site infections in orthopedic patients in an orthopedic trauma clinical medical center in shanghai. Surg Infect (Larchmt).

[23] Kutter E (2009). Phage host range and efficiency of plating. Methods Mol Biol.

[24] O’Flaherty S, Coffey A, Edwards R, Meaney W, Fitzgerald GF, Ross RP (2004). Genome of staphylococcal phage K: a new lineage of myoviridae infecting gram-positive bacteria with a low G + C content. J Bacteriol.

[25] Gu J, Liu X, Lu R, Li Y, Song J, Lei L, Sun C, Feng X, Du C, Yu H (2012). Complete genome sequence of *staphylococcus aureus* bacteriophage GH15. J Virol.

[26] Darling AE, Tritt A, Eisen JA, Facciotti MT (2011). Mauve assembly metrics. Bioinformatics.

[27] Tamura K, Peterson D, Peterson N, Stecher G, Nei M, Kumar S (2011). MEGA5: molecular evolutionary genetics analysis using maximum likelihood, evolutionary distance, and maximum parsimony methods. Mol Biol Evol.

